# Early Clinical and Radiographic Results of Minimally Invasive Anterior Approach Hip Arthroplasty

**DOI:** 10.1155/2014/954208

**Published:** 2014-03-02

**Authors:** Tamara Alexandrov, Elke R. Ahlmann, Lawrence R. Menendez

**Affiliations:** ^1^Department of Orthopaedics, Los Angeles County-University of Southern California Medical Center, 1200 N. State Street, GNH 3900, Los Angeles, CA 90033, USA; ^2^University of Southern California, Keck School of Medicine, Los Angeles County-University of Southern California Medical Center, 1200 N. State Street, GNH 3900, Los Angeles, CA 90033, USA; ^3^University of Southern California, Keck School of Medicine, USC University Hospital, 1510 San Pablo Street, Suite 634, Los Angeles, CA 90033-4608, USA

## Abstract

We present a retrospective review of the early results and complications in a series of 35 consecutive patients with 43 total hip arthroplasties performed through an anterior muscle sparing minimally invasive approach. We found the early complication rates and radiographic outcomes comparable to those reported from arthroplasties performed via traditional approaches. Complications included dislocation (2%), femur fracture (2%), greater trochanteric fracture (12%), postoperative periprosthetic intertrochanteric fracture (2%), femoral nerve palsy (5%), hematoma (2%), and postoperative iliopsoas avulsion (2%). Radiographic analysis revealed average cup anteversion of 19.6° ± 6.6, average cup abduction angle of 48.4° ± 7, stem varus of 0.9° ± 2, and a mean leg length discrepancy of 0.7 mm. The anterior approach to the hip is an attractive alternative to the more traditional approaches. Acceptable component placement with comparable complication rates is possible using a muscle sparing technique which may lead to faster overall recovery.

## 1. Introduction

Despite advances in implants and greater understanding of hip biomechanics, complications such as dislocation, abductor weakness, leg length discrepancy, and component malpositioning continue to plague recipients of total hip arthroplasties [1–14]. In attempts to circumvent these complications many investigators have recently turned to the anterior approach first described by J. Judet and R. Judet in 1950 [15–21]. It has been referred to as a minimally invasive technique that allows exposure of the proximal femur and acetabulum through a small incision without the need to cut muscles or perform osteotomies. Theoretically, this should lead to shorter recovery time and fewer major complications that have been associated with the more traditional approaches.

Minimally invasive total hip arthroplasty has recently become popular in attempts to decrease recovery time and improve cosmesis. Shorter operative times and faster return to normal gait have been noted in some studies [22–24]; however, others found no difference in functional outcome scores, early function, hospital stay, and intraoperative blood loss [23–27]. Another concern involves accurate component placement as the smaller incision lends itself to poor visualization and cemented stems placed through a minimally invasive incision may have a propensity for varus positioning and a higher incidence of acetabular component malposition [25, 27].

The purpose of this study was to evaluate our early intraoperative and postoperative experience with the anterior minimally invasive approach to total hip arthroplasty and to compare the clinical results to those reported for traditional approaches. Postoperative radiographs were also reviewed to determine whether accurate component placement and leg length equality can be achieved with this technique.

## 2. Materials and Methods

We performed a retrospective review of the first 43 consecutive primary hip arthroplasties in 35 patients performed by a single surgeon via the anterior minimally invasive approach at our institution between 2002 and 2008. We began performing the anterior approach in 2002 and have since then exclusively used this technique for all primary total hip procedures. Eight of these patients underwent bilateral total hip arthroplasties, and, of these, five patients had both hips operated on the same day. There were 14 male and 29 female patients with a mean age of 62 years (range, 39 to 83). Twenty-four of the arthroplasties performed were on the left hip and 19 were performed on the right one. The average body mass index (BMI) was 26 (range, 18 to 37). Patients had an average of 2 (range, 0 to 4) reported medical problems, among the most common being hypertension and hypothyroidism. The etiology of the hip disease was degenerative arthritis in 29 hips, avascular necrosis in eight hips, and rheumatoid arthritis in six hips. One of the patients with degenerative joint disease had a previously undiagnosed femoral neck fracture on presentation and one had a history of pigmented villonodular synovitis. Two of the patients with rheumatoid arthritis had large benign cystic masses in their hips that were resected at the time of surgery. The average length of followup was 16.8 months (range, 12 to 60.7 months).

All operations were performed by the senior author (LRM). The surgical technique utilized is similar to other published reports [18, 19]. The Stryker MIS system (Stryker, Rutherford, NJ, USA) was used in the first 18 patients and the Corail system (DePuy Orthopaedics, Warsaw, IN, USA) was used in the last 25.

**Figure 1:**
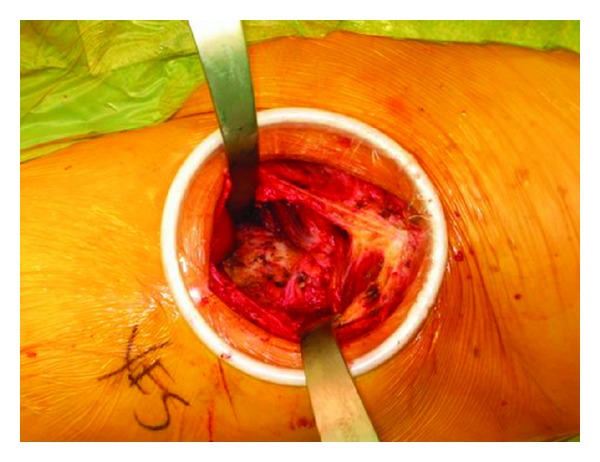
Exposure of the joint capsule seen through the Dexterity Protractor ring (Dexterity Surgical Inc., Roswell, GA, USA) which self-retracts and protects the skin and soft tissues.

The patient was placed in supine position on the PROfx table (Mizuho OSI, Union City, CA, USA) and the operative extremity draped such that the limb could be manipulated freely. An incision 8 cm in length was made beginning at a point 1 cm distal and 2 cm lateral to the anterior superior iliac spine and heading in a longitudinal/oblique direction towards the greater trochanter. Once the skin incision had been made and the fascia over the tensor fascia lata reached, full thickness flaps were elevated. A self-retaining Dexterity Protractor retractor sleeve (Dexterity Surgical Inc., Roswell, GA, USA), as has been popularized in abdominal surgery, [28] was inserted within the incision to allow for maximal retraction without damaging the surrounding skin (Figure 1). The fascia overlying the tensor fascia lata was incised in line with the skin incision, taking great care to protect the lateral femoral cutaneous nerve. The interval between the tensor fascia lata muscle and the sartorius was then identified and bluntly developed, as was the deeper interval between the gluteus medius and the rectus femoris. The ascending branch of the lateral femoral circumflex artery found in the distal portion of this interval was ligated. The reflected head of the rectus femoris muscle was then identified and divided from the superior lip of the acetabulum. The anterior joint capsule was then visualized.

An anterior capsulectomy was performed to access the hip joint. We have found capsulectomy to be preferable to capsulotomy as the divided capsule otherwise interferes with visualization and cup placement. Once the capsule was incised, the femoral head and neck were exposed by two Hohmann retractors placed on either side of the neck. In order to aid with removal of the head, the hip was first partially dislocated to loosen soft tissue attachments. This was done by placing manual traction and externally rotating the leg spar. After this, traction was then released, and the neck cut was made with an oscillating saw. A corkscrew instrument was then placed into the femoral head and the head was removed with the use of a skid. Long curved scissors were used to divide the ligamentum teres.

**Figure 2:**
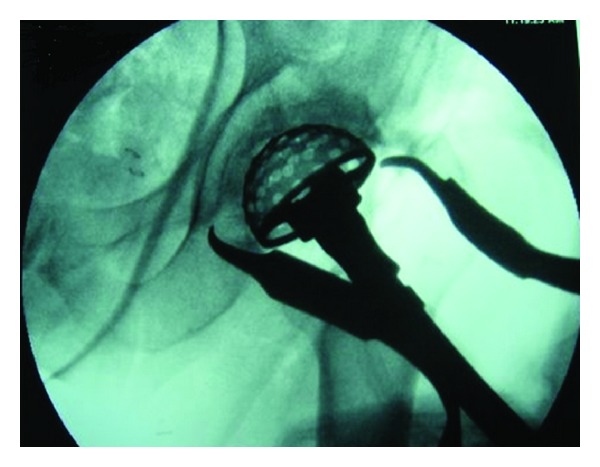
Preparation of the acetabulum with reamers is performed under fluoroscopic guidance.

After the head was removed, attention was turned to the acetabulum. The Hohmann retractors were placed over the anterior and posterior rims of the acetabulum for exposure. The labrum was cleared from the edges of the acetabulum as were any overriding osteophytes and excess tissue. After visualization was achieved the acetabulum was reamed to the appropriate size. At this time intraoperative fluoroscopic imaging was used to confirm appropriate placement of the reamers and trials (Figure 2). Our goal for acetabular component placement was abduction of 40–45 degrees and anteversion of 15–20 degrees. The live components were then placed in the acetabulum. In this series, 41 acetabular cups were press fit and two were cemented. Nineteen of the press fit cups were additionally fixated with screws.

**Figure 3:**
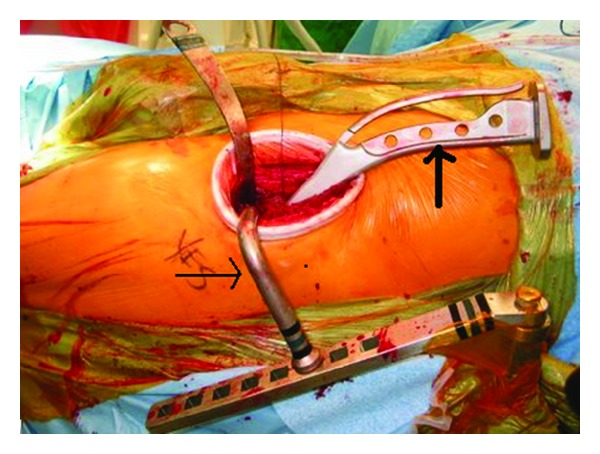
Intraoperative photograph illustrating the use of the femoral hook attachment (thin arrow) and curved broach handle (thick arrow), used to prepare the femur.

Attention was then directed towards the femur, where the femoral hook attachment was placed around the proximal femur. The hook attachment allowed the femur to be delivered anteriorly and was controlled by a jack at the head of the table (Figure 3). Excessive anterior force must be avoided as this risks fracture of the greater trochanter. The leg was then externally rotated, extended, and adducted to gain maximal exposure of the proximal femur. The lateral gutter of the femur was cleared of excess tissue to allow component placement without varus. Specially shaped broach handles were used to gain exposure to the intramedullary canal and the femur was broached to the appropriate size. Fluoroscopy was again used to confirm trial component placement. Once the femoral component was inserted the trial femoral head was placed. In order to locate the hip, the femoral hook was lowered and the leg brought into traction and internal rotation using the leg spar. Stability was then assessed by placing the leg in a neutral position and slowly rotating it to 90 degrees of external rotation. If stable, the hip should remain reduced when the femur is pulled laterally with a hook placed around the neck of the femoral component. Leg length discrepancy was also determined by performing an AP of the pelvis with fluoroscopy and comparing the positions of the lesser trochanters.

After live component placement, all wounds were closed in the usual manner and a suction drain was inserted in the hip joint. The patients received antibiotics until the drain was removed once the output had decreased to <50 mL/day, and all patients were placed on pharmacologic DVT prophylaxis for six weeks postoperatively. Patients were seen by the physical therapist on the first postoperative day and encouraged to ambulate with full weight without the use of a brace. Patients were restricted only to anterior hip precautions which consisted of no external rotation with the hip in full extension. They were otherwise given no other restrictions as to motion or activities. Patients were discharged from the hospital when becoming medically stable. Routine followup occurred postoperatively at 6 weeks, 3 months, 6 months, 1 year, and annually thereafter.

The patients' clinical records were reviewed for operative time, intraoperative complications, estimated blood loss, surgical technique, length of admission, postoperative course, and complications. The findings were compared to historical controls from the existing literature.

Postoperative radiographs were reviewed to determine acetabular anteversion, acetabular cup abduction, femoral stem varus positioning, and leg length discrepancy. Acetabular component anteversion was calculated as the arcsin of the short axis of the ellipse formed by the cup divided by the longer axis [29, 30]. Acetabular inclination was taken as the angle between a line drawn between the teardrops and the long axis of the acetabular ellipse [29, 31–33]. The distance between a point chosen on the lesser trochanters and a line drawn between the ischial tuberosities was used to determine leg length discrepancy. All calculations and measurements were performed by a single orthopaedic surgeon (TA).

## 3. Results

Operation time averaged 146 minutes (range, 100 to 245) for unilateral hips, 278 minutes (range, 205 to 352) for bilateral hips, and 176 minutes (range, 110 to 352) for all hips combined. The mean intraoperative blood loss was 1157 mL (range, 410 to 2600), and patients received an average of 2 units (range, 0 to 4) of packed red blood cells intraoperatively. Postoperatively they received a mean of 1 unit (range, 0 to 3) in addition to that given during surgery. The average hospital stay for unilateral hips was 5.3 days (range, 2 to 8 days) and 8.4 days (range, 3 to 14 days) for bilateral hips. When comparing the data from our first ten cases to our last ten cases we found an improvement in surgical time and intraoperative blood loss, indicating that these parameters improve with surgeon experience (Table 1).

Intraoperative complications included two fractures (5%, 2/43) (Table 2). One femoral shaft fracture occurred which was stabilized with cables as well as one greater trochanteric fracture that was fixed with cerclage wires. The femoral fracture occurred in a patient with severe osteopenia and avascular necrosis of the proximal femur. When the contralateral hip arthroplasty was performed at a later time, the femoral shaft was prophylactically secured with cables to prevent a similar occurrence.

Six fractures were reported in the postoperative period (14%, 6/43). Five minimally displaced fractures of the greater trochanter were noted during postoperative followup. All were amenable to nonoperative treatment. One patient returned to the operating room postoperatively for internal fixation of a periprosthetic intertrochanteric fracture.

There were no infections or wound dehiscences in this series. One patient developed a deep hematoma that was surgically evacuated one week after the primary procedure. This patient had been treated with anticoagulants prior to surgery for a remote history of pulmonary embolism.

Two documented femoral nerve palsies were noted during the follow-up period, both of which were confirmed with EMG. All symptoms resolved in both patients within three months of surgery. In addition, one postoperative iliopsoas avulsion was diagnosed clinically and treated with physical therapy with subsequent improvement of symptoms.

One hip dislocation occurred in this series six weeks after surgery (2%, 1/43) after a fall. A successful closed reduction was performed and the patient was placed in a hip abduction brace for six weeks. Upon 17 months of followup the patient has not had any recurrent episodes of instability.

Acetabular and femoral component placement with the anterior approach was measured on plain radiographs (Table 3). Of the 43 hips operated, 41 had radiographs available for review. The average acetabular anteversion was 19.6° ± 6.6° (range, 5° to 30°). When both AP pelvis and AP hip radiographs were available (31 hips), anteversion was confirmed by comparing the two [29, 30, 34]. In the cases where only one of the two views was available (10 hips), anteversion was assumed. The acetabular component version was calculated on the AP of the hip when it was available. Otherwise, the AP pelvis was used. Since acetabular anteversion appears smaller on radiographs centered on the pubis [35], this may have caused our average to be underestimated. The average acetabular cup abduction was found to be 48.4° ± 7° (range, 34° to 70°). Fifteen hips had abduction of greater than 50 degrees. The mean stem varus was found to be 0.9° ± 2° (range, −4° to 6°). The average leg length discrepancy was 7 mm (range, 3 to 14 mm).

## 4. Discussion

The main benefits of the anterior approach are fast recovery and improved early patient function. In our experience, almost all patients achieve full weight bearing by the first postoperative day, and most are able to ambulate without the use of crutches by the time of their hospital discharge. None of the patients in this series ambulated with a postoperative limp or had any evidence of muscle weakness often seen with the posterior and lateral approaches to the hip where the incidence of limp has been documented in up to 20% of patients [1, 2]. This is primarily attributable to the fact that no muscles are cut or sustain extensive damage during the anterior approach [36].

Dislocation rates for total hip arthroplasty range from 0.3% to 11% [1–10, 37, 38]. Rates of dislocation with the posterior hip approach are higher than those reported for lateral and anterolateral approaches [6, 7, 13]. This may be due to several factors including violation of the posterior capsule, division of the short external hip rotators, and component positioning. A study comparing the dislocation rate between capsulectomy and capsular repair with a posterolateral approach reported 2.8% dislocations with capsulotomy, while repair of the capsule resulted in a significantly lower rate of 0.6% dislocations [3]. Previous studies on the anterior hip approach have reported a dislocation rate ranging between 0% and 2% [18, 19, 21, 39, 40]. Our dislocation rate of 2% falls within this range.

Another factor associated with dislocation may be component malpositioning [4, 5, 37, 38]. The more common surgical approaches require that the patient be positioned in the lateral decubitus position which may result in tilting of the pelvis anteriorly during surgery [11, 12]. This anterior pelvic tilt if unrecognized intraoperatively may result in placement of the acetabular cup with inadequate anteversion, thus predisposing to posterior dislocation. This may be avoided with the anterior approach which appears to allow for reproducible and accurate component placement. In a study of 100 cadavers, the average acetabular anteversion was found to be 19.9° ± 6.6 [41]. Our calculated cup anteversion of 19.6° ± 6.6 is well within the recommended “safe zone” of 5 to 25 degrees [4] and is comparable to physiologic measurements [32, 42–45]. For our calculations we used two separate methods to calculate the angle of anteversion [29, 30, 43] and both methods independently obtained a value of 19.6° ± 6.6. We therefore believe that our results are as accurate as possible and are comparable to the only other previous study reporting cup anteversion values of 19.4° ± 5.2 [18]. Our mean cup abduction angle of 48.4° ± 7, leg length discrepancy of 7 mm, and stem varus of 0.9° ± 2 are all within recommended guidelines [4, 41] and are similar to those findings reported by other investigators with this technique [18–20].

Another notable complication in our series was a 14% (6/43) rate of greater trochanteric fracture, which is similar to that reported in a large multicenter study of 1,152 patients in which 12% sustained greater trochanter fractures. Of the fractures in our series, one occurred intraoperatively and was secured with cables, and 5 were noted postoperatively. The 5 postoperative fractures, none of which required surgery, represented either small avulsions or were nondisplaced. We believe that these fractures may have occurred either during elevation of the femoral hook component of the table when excessive anteriorly directed force may have caused avulsion or fracture of the greater trochanter in osteoporotic patients or during the reduction maneuver required to relocate the hip during trialing and final hip relocation. During broaching the femur is in a shortened, adducted, extended, and externally rotated position. When the hip is then reduced, if abduction is performed prior to traction, internal rotation, and flexion, the greater trochanter abuts the ilium and may easily fracture. This was observed intraoperatively in the one case requiring cable fixation. It is the recommendation that the reduction maneuver be performed initially with traction, internal rotation, flexion back to neutral, and lastly abduction, and this must be emphasized to the assistant in control of the leg spars. The ankle injuries apparently caused by intraoperative traction reported in previous studies [18, 21] were not encountered in our series.

Two femoral nerve palsies occurred in our series, both of which resolved within three months postoperatively. We believe that these may have occurred when the hip was placed in a hyperextended position using the leg spar of the table during femoral canal preparation. The femoral nerve is susceptible to traction injury during this time if the hip is placed in excessive extension. Studies have shown that extensive hip abduction and external rotation may also be associated with femoral nerve traction injury [40, 46, 47]. Care must be taken when performing the anterior approach to assure that the hip is not in a position of excessive abduction and extension placing excessive tension and traction on the femoral nerve.

There does appear to be a rather steep learning curve when performing this procedure as our operative time and our blood loss significantly improved with time and additional surgeon experience as seen when comparing our first ten to our last ten cases. This was also found in a large multicenter observational study, where surgeons who had performed less than 100 cases were twofold more likely to have complications in their patients [48]. Another series defined the learning curve to be around 40 cases, or 6 months in a high volume hip arthroplasty center, after which operating time and blood loss stabilize, and approach-related complications can be avoided [49].

The anterior minimally invasive muscle sparing approach to the hip is an attractive alternative to the more traditional posterior, lateral, and anterolateral approaches. From our early results we can conclude that the incidence of early dislocation is not increased and that accurate reproducible component placement is possible. As with any new technique, a period of trial and error is necessary to identify weaknesses in the approach and to develop new technical advances. In this series of our first 43 cases, we have demonstrated that there is a learning curve with this procedure and care must be taken to avoid certain pitfalls and complications which are specific to the instrumentation and technique of the anterior approach. Further studies are necessary to objectively examine whether the anterior minimally invasive approach provides advantages in terms of recovery time and long-term functional outcome in comparison to other approaches. We believe that as it gains popularity, this could become a superior approach with advantages for both surgeon and patient.

## Figures and Tables

**Table 1:** Comparison of intraoperative and postoperative data for first ten unilateral versus last ten unilateral hip cases.

	First 10 cases	Last 10 cases
Surgical time (average minutes)	229.5 (range, 165–315)	139 (range, 100–170)
Intraoperative blood loss (average milliliters)	2180 (range, 600–2600)	500 (range, 250–900)
Hospital stay (average days)	4.5 (range, 2–8)	4.4 (range, 3–9)
Complications	4	3

**Table 2:** Intraoperative and postoperative complications following anterior approach total hip arthroplasty.

Complication	Number of hips	Complication rate
Intraoperative		
Femoral fracture	1	2.3%
Greater trochanter fracture	1	2.3%
Postoperative		
Greater trochanter fracture	5	11.6%
Femoral nerve palsy	2	4.6%
Intertrochanteric fracture	1	2.3%
Iliopsoas avulsion	1	2.3%
Dislocation	1	2.3%
Hematoma	1	2.3%

**Table 3:** Postoperative radiographic measurements.

Radiographic measurement	Average value	Range
Acetabular anteversion	19.6° ± 6.6°	5° to 30°
Acetabular abduction	48.4° ± 7°	34° to 70°
Stem varus	0.9° ± 2°	−4° to 6°
Leg length discrepancy	7 mm	3 to 14 mm
